# SNP-Based and Kmer-Based eQTL Analysis Using Transcriptome Data

**DOI:** 10.3390/ani14202941

**Published:** 2024-10-11

**Authors:** Mei Ge, Chenyu Li, Zhiyan Zhang

**Affiliations:** National Key Laboratory for Swine Genetic Improvement and Germplasm Innovation Technology, Jiangxi Agricultural University, Nanchang 330045, China; gmei15797692660@gmail.com (M.G.); 18702523671li@gmail.com (C.L.)

**Keywords:** RNA-seq, eQTL, SNP, kmer, haplotype block

## Abstract

**Simple Summary:**

Expression quantitative trait locus (eQTL) analysis is crucial in revealing the genetic basis of complex traits, advancing the study of human diseases, optimizing the breeding of agricultural plants and animals, and gaining a deeper understanding of specific biological processes. The conventional analysis involves correlating genetic variants from whole-genome sequencing (WGS) data and gene expression, but to improve the power of eQTL detection, it is often necessary to expand the sample size as far as possible, ranging from hundreds to thousands. Large sample sizes for WGS are extremely costly, and this bottleneck is particularly evident in economically important agricultural animals. In contrast, the advantages of eQTL analyses from transcriptome data are obvious: cost-effective, simultaneous acquisition of variants and expression information. We propose to use transcriptome data alone for SNP calling and kmer generation, and then association analysis with gene expression. Here, 87 SNP-based and 35 kmer-based association results were obtained. Subsequently, comparison and validation of these two results revealed that they each have their own strengths and can complement each other, which promotes in-depth exploration of the regulatory relationship between genetic variants and gene expression.

**Abstract:**

Traditional expression quantitative trait locus (eQTL) mapping associates single nucleotide polymorphisms (SNPs) with gene expression, where the SNPs are derived from large-scale whole-genome sequencing (WGS) data or transcriptome data. While WGS provides a high SNP density, it also incurs substantial sequencing costs. In contrast, RNA-seq data, which are more accessible and less expensive, can simultaneously yield gene expressions and SNPs. Thus, eQTL analysis based on RNA-seq offers significant potential applications. Two primary strategies were employed for eQTL in this study. The first involved analyzing expression levels in relation to variant sites detected between populations from RNA-seq data. The second approach utilized kmers, which are sequences of length k derived from RNA-seq reads, to represent variant sites and associated these kmer genotypes with gene expression. We discovered 87 significant association signals involving eGene on the basis of the SNP-based eQTL analysis. These genes include *DYNLT1*, *NMNAT1*, and *MRLC2*, which are closely related to neurological functions such as motor coordination and homeostasis, play a role in cellular energy metabolism, and function in regulating calcium-dependent signaling in muscle contraction, respectively. This study compared the results obtained from eQTL mapping using RNA-seq identified SNPs and gene expression with those derived from kmers. We found that the vast majority (23/30) of the association signals overlapping the two methods could be verified by haplotype block analysis. This comparison elucidates the strengths and limitations of each method, providing insights into their relative efficacy for eQTL identification.

## 1. Introduction

Genome-wide association studies (GWAS) have identified thousands of candidate SNP loci associated with complex traits or diseases. Since most of these loci are located in non-coding regions, research on their regulatory mechanisms have not been fully explored [1,2]. It is generally assumed that the SNP loci are associated with specific traits by affecting gene expression and thus function, which is known as eQTL (expression quantitative trait locus) analysis [3]. eQTL analyses have been extensively studied in human diseases, such as Type 2 diabetes [4], chronic kidney disease [5], and metabolic diseases [6], and can help identify the causative genes and understand the molecular mechanisms of disease development. Similarly, it also has considerable application value in agricultural animals and plants, where it can be applied to breeding by identifying eQTLs associated with economic traits such as meat quality [7,8], growth and carcass traits [9], disease resistance [10], etc., which can improve production performance.

Traditional eQTL mapping uses SNPs identified by whole-genome sequencing (WGS) data and gene expression levels obtained from transcriptome data for association analysis. Although the density of SNPs identified by WGS is higher, the cost of sequencing has greatly increased due to the need for a large enough sample size, even in the context of the decline in the sequencing costs. In contrast, RNA-seq data are more readily available, and not only can they provide information on gene expression and the SNPs in genes, but the cost of sequencing is also lower. Therefore, eQTL analysis based on transcriptome data has important potential applications.

The integrative analysis of eQTLs and GWAS has been widely reported and can help uncover the regulatory mechanisms underlying complex traits of interest [11,12]. Generally, there are two strategies for GWAS analysis. One is to detect variant loci among large-scale populations and then conduct an association analysis between gene expression levels and genetic variants. SNPs are the most common form of genetic variations in the genome, and weak association signals with specific traits can be detected through the study of large-scale samples [13]. Although the detection of SNPs has become more diverse and efficient with the development of genomics, their identification is highly dependent on the reference genome, which can lead to inaccurate genotyping in species with poor quality reference genomes. Therefore, GWAS analysis based on kmers has emerged. Sequences of length k, known as kmers, are extracted from sequencing data, and then association analyses of the genotypes of the kmers and gene expression are performed. Unlike SNPs that contain only one variant type, kmers can theoretically contain any variant type, such as SNPs, insertions, deletions, inversions, and translocations [14] (Supplementary Figure S1). In addition, this method can be effectively applied to species without a reference genome or with a large number of errors in the reference genome [15]. Currently, the GWAS method based on kmers has been widely studied in plants and bacteria [16,17], with limited research in animals.

**Figure 1:**
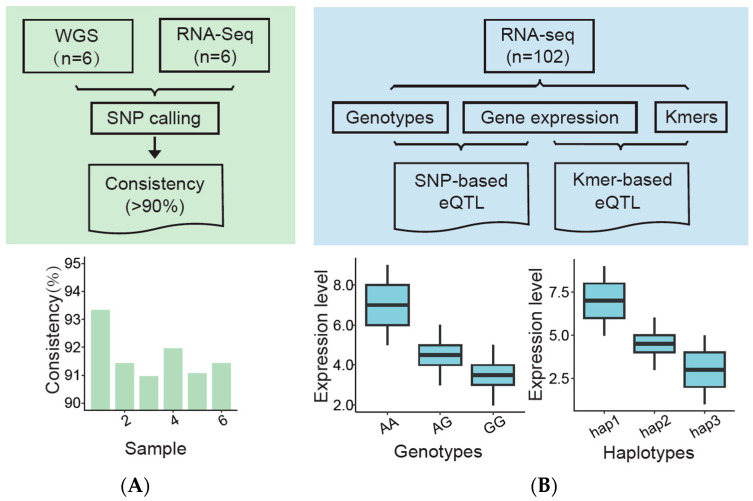
Schematic diagram of the study design. (**A**) SNP consistency checkup using WGS and RNA-seq data (left panel, green); (**B**) assessment of the two methods of eQTL analysis (right panel, blue).

In this study, we first compared the consistency of variants identified from WGS and RNA-seq data. We hypothesized that these SNPs would have a high degree of consistency, and we then provided two different methods to identify candidate variant loci that affect gene expression level. One was based on the SNPs identified by RNA-seq data and gene expression for eQTL analysis. The other was performed based on kmers obtained from RNA sequencing reads and gene expression (Figure 1). It is worth noting that the two methods proposed in this study demonstrate a novel perspective for identifying eQTLs, which is crucial for unearthing the regulatory mechanisms of the candidate causal loci or genes underlying complex traits and diseases.

## 2. Materials and Methods

### 2.1. Stage of Identifying SNPs’ Consistency Using DNA/RNA

#### 2.1.1. Identification of Variants via RNA Sequencing

Muscle tissue from six pigs approximately 90–100 days old was collected after slaughter. The samples were snap-frozen in liquid nitrogen and stored at −80 °C, and then used for RNA sequencing. Raw RNA-seq reads were processed using fastp v0.23.2 [18]. The clean reads were aligned to the susScr11 (Sus scrofa 11.1) reference genome using STAR v2.7.3 [19], and the mapped reads were sorted and indexed with samtools v1.9 [20]. The sequence mapping rate of the reads was summarized using the samtools flagstat command. To mark duplicate reads, sambamba v0.8.2 [21] was employed. Next, fragments of reads falling in intronic intervals were directly excised and the MAPQ was adjusted using GATK SplitNCigarReads v4.1.4.1 [22]. Subsequently, GATK AddOrReplaceReadGroups and HaplotypeCaller were used to add group information and call variants for each sample, respectively. GVCFs files from different individuals were combined, and a VCF (variant call format) file containing information on variations for multiple individuals was generated with GATK CombineGVCFs and GenotypeGVCFs [22]. The SNP (single nucleotide polymorphism) variants were then retained. After rigorous filtering, high-quality variant information was used for downstream analyses.

#### 2.1.2. Identification of Variants via Whole-Genome Sequencing

DNA sequencing data of ear tissue from the six pigs mentioned above were used. Raw sequencing reads obtained from WGS were processed using fastp v0.23.2 [18], and the clean reads were mapped to the susScr11 reference genome using BWA MEM v0.7.17 [23]. Subsequently, the mapped reads were sorted by samtools v1.9 [20]. To add group information to the samples and mark duplicate reads, GATK AddOrReplaceReadGroups and MarkDuplicates [22] were employed, respectively. For each sample, variants were called with GATK HaplotypeCaller. Next, after the same process as described above for the RNA-seq data, the final high-quality variant information from the VCF file was obtained.

#### 2.1.3. Comparison of Variants Identified via RNA-seq and WGS

The high-quality variants identified via RNA-seq and WGS were compared by the following steps. Briefly, the genomic coordinates and genotype information of the variants from the VCF file were extracted using the bcftools v1.13 [24] query command. To circumvent the influences of some potential confounding factors, a concordance analysis was conducted using a specific set of autosomal variants that are also located on exons. This analysis helped to assess the reliability of the SNP-based and kmer-based association analyses using genetic variants (e.g., SNPs) identified by RNA sequencing data.

### 2.2. Stage of eQTL Analysis Using RNA-seq Data

#### 2.2.1. RNA Sequencing Data and Variant Calls

The RNA sequencing data of muscle tissue from 102 pigs were randomly downloaded from EBI ENA (accession numbers PRJEB28465, PRJNA344688, PRJNA386796, PRJNA403969, PRJNA506343, and PRJNA515765) (Supplementary Table S1). The raw sequencing reads were trimmed and filtered by fastp v0.23.2 [18], and the clean reads were mapped to the susScr11 reference genome using HISAT2 v2.2.0 [25]. With samtools v1.9 [20], the mapped reads were sorted and indexed. Gene expression levels (both counts and TPM) were calculated using StringTie v2.2.1 [26]. Genes that had TPM values > 1 were considered to be expressed. Subsequently, genes with count numbers > 6 and TPM values > 1 in at least 80 samples were retained. In total, 12,660 genes were selected after stringent filtering criteria. TPM values per gene were then quantile-normalized and inverse normal transformed with the R package RNOmni [27]. The expression levels of these genes were used as molecular phenotypes for SNP-based and kmer-based expression quantitative trait locus (eQTL) analyses.

The sorted bam files were used for the downstream variant calling. To save the computational load and time consumed, using the Graphtyper v2.7.5 [28] genotype and vcf_concatenate, a VCF file with the variation information for each individual was created and data on variants from different samples were combined. The final variants from the RNA-seq data were used as genotype files for downstream SNP-based association analyses.

#### 2.2.2. Phenotypes and Covariates

After screening and processing, the expression levels (TPM values) of the aforementioned genes were regarded as the raw phenotypes. Phenotypes were then corrected using the first three principal components of the genotype, sex, slaughter age and tissue subcategories using the R package v4.3.3 (R: The R Project for Statistical Computing (r-project.org)).

#### 2.2.3. Genotypes and Constructing Kinship Matrices

Genotypes and kinship matrices for SNP-based association analysis were obtained according to the following steps. The VCF files derived from the RNA-seq data were first transformed into PLINK binary files using plink v1.9 [29]. Then the minor allele frequency and the calling rate of the SNP genotypes were filtered. Using plink, SNPs were selected according to their linkage imbalance and extracted. Subsequently, the kinship matrix was constructed using gemma v0.98.5 [30] according to the default parameters. The kinship matrix obtained in this step was also employed for kmer-based association analysis.

The following steps were followed in order to obtain genotypes for kmer-based association analysis. Briefly, 31 bp kmers were generated from the clean sequencing reads for each sample using kmc v3.2.1 [31]. To avoid kmers caused by sequencing errors, only kmers that occurred at least twice were included in the downstream analyses. The intersection and union of the kmers for all samples were generated using kmc_tools v3.2.1 [31]. The kmers_subtract function of kmc_tools was used to select the set of differences of the two sets of kmers. Next, the intersection between the set of kmers for each sample and the set of differences was calculated, and then the union with the set of differences was taken. The binary files of the kmers were converted into readable text files using the transform function of kmc_tools. After that, 30 samples were randomly selected to perform the union operation of the kmers, and then the set of kmers of each sample was employed for the intersection operation with the union sets. The frequency distribution of the kmers was checked through the kmer histogram, and the kmer counts of each sample were obtained through calculation. Finally, the kmer counts were integrated to obtain the final kmer counts for all samples.

#### 2.2.4. Association Analysis and Annotation of Kmers

The kmer-based association analyses were performed using univariate linear mixed models with Gemma v0.98.5 [30], similar to conventional GWAS studies. Meanwhile, the association results were preliminarily filtered on the basis of the the minor allele frequencies (MAFs) and P-values. In order to explore the biological functions of the retained kmers from the RNA sequencing reads, genomic localization of the kmers was performed. The kmers were aligned to the susScr11 reference genome using bowtie2 v2.5.1 [32] and then sorted and indexed using samtools v1.9 [20]. It is worth noting that the mapped results were also carefully screened on the basis of the mapping quality (MAPQ) in order to avoid the effects of genomic repeat regions and sequencing errors. Finally, the kmer-based association results and genomic coordinate results were integrated.

#### 2.2.5. Validation Analysis

To obtain reliable significant signals, the association results were processed as follows. First, a correlation analysis was performed between the genotypes of the top SNP sites and the expression levels in all samples using the ANOVA test and filtered with the *p*-value. In addition, the variants from the RNA-seq data filtered by the minor allele frequency and genotype calling rate were labeled according to the default parameters (-t AN AC) using the +fill-tags function of bcftools v1.13 [24]. Then, the aforementioned VCF file was used to infer the phase origins using the shapeit5 v5.1.1 [33] phase_common_static command. The BCF format files were transformed to VCF format using the bcftools v1.13 [24] convert command and then compressed and indexed using bgzip v1.9 and tabix v1.9 [34], respectively. Next, a correlation analysis between the haplotype blocks and gene expression was carried out through a customized shell script, in which the haplotype blocks consisted of the top SNP sites and 10 SNP sites on each of their flanks, while only haplotype blocks with more than 10 counts were retained for further analysis. Moreover, the final significant signals were searched in the public piggtex website (PigGTEx-Portal (farmgtex.org)) to check their robustness.

## 3. Results

### 3.1. Consistency of SNPs Identified by WGS and RNA-seq

**Figure 2:**
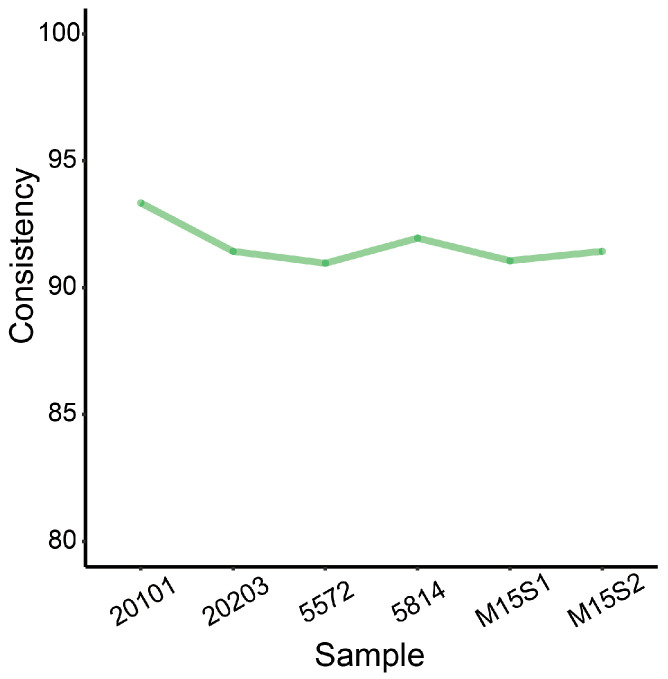
Comparison of the consistency of SNPs identified by WGS and RNA-seq. The *x* axis indicates the name of the sample, and the *y* axis indicates the percentage of consistency.

On the basis of an understanding of traditional expression quantitative trait locus (eQTL) mapping, we aimed to explore whether eQTL mapping could be performed using only transcriptome data. First, to compare single nucleotide polymorphism (SNP) variants identified from WGS and RNA-seq, we collected WGS data from ear tissue and RNA-seq data from the muscle tissue of six pigs from an existing database of our laboratory. Next, following quality control, on average, 983.8 million clean reads were produced per sample from the WGS data, of which 948.7 million could be precisely mapped to the susScr11 reference genome, accounting for more than 96% (Supplementary Table S3). Genomic variants were identified using GATK HaplotypeCaller [22], and in total, 24,872,169 high-quality SNP loci were retained after stringent screening criteria. In the RNA-seq data, 1528 million clean reads in total were generated after quality control, of which a total of 1387 million reads could be perfectly aligned (Supplementary Table S2). After the same process, 3,238,240 highly reliable variants were retained at the transcriptome level. Subsequently, 905,513 and 267,000 variants and expression variants (eVariants) located in the exon region were extracted, respectively. The proportion of eVariants from the transcriptome level that overlapped with variants from the genome level was 96.83%. Further comparing these overlapped variants showed that, on average, their SNPs’ consistency was about 92% in each individual (Figure 2). These results suggest that the basis of our study is highly credible.

### 3.2. SNP-Based eQTL Analysis Using Transcriptome Data

**Figure 3:**
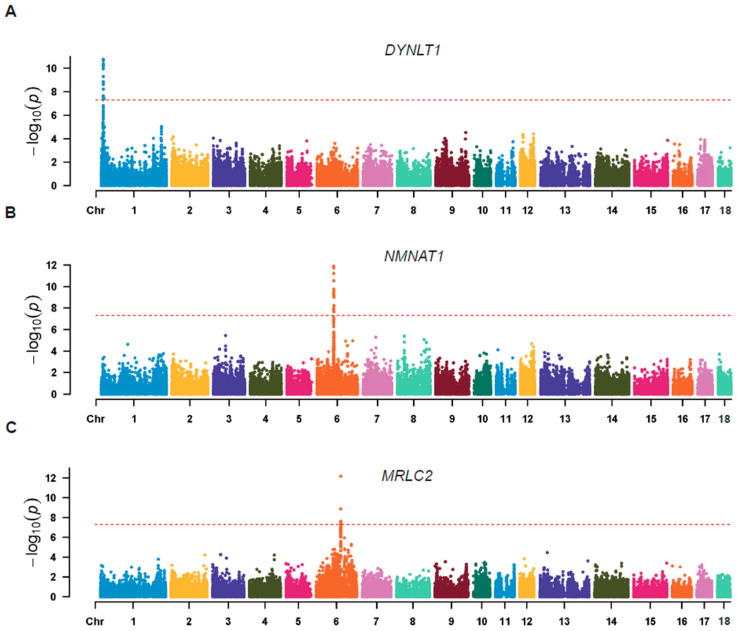
Manhattan plots of the association results of the representative genes. Manhattan plots of (**A**) *DYNLT1*, (**B**) *NMNAT1*, and (**C**) *MRLC2*. The red dashed line in the Manhattan plots represents the level of significance.

RNA sequencing data from 102 pig muscle tissues were downloaded from the publicly available PigGTEx database (PigGTEx-Portal (farmgtex.org)) for downstream eQTL analysis. After variant calling, in total, 1,045,504 SNPs loci were initially generated, and 76,627 and 768,682 variants were filtered using PLINK [29] according to the minor allele frequency and genotype calling rate (--maf 0.03 --geno 0.1), respectively. Following quality control, 200,195 independent variants were retained as genotypes for the association analysis. At the same time, to avoid population stratification, these variants were used in principal component analysis (PCA), where the first three principal components were used to correct the population structure (Supplementary Figure S2). These SNPs were then screened and extracted according to linkage disequilibrium (LD), which was used to construct the kinship matrix. For the gene expression levels, 35,670 genes were quantified using StringTie [26]. Moreover, 12,660 genes were retained using gene counts and TPM value filtering criteria, followed by quantile normalization and inverse normal transformation, as well as correction for various batch effects. According to the association analysis between reliable SNPs and gene expression, 87 association signals were found at a significant *p*-value of 5 × 10^−8^. We performed cis-eQTL analysis using MatrixEQTL (pvOutputThreshold_cis = 1 × 10^−5^, cisDist = 1 Mb) and found that 76 of the 87 significant association results overlapped. This high proportion further illustrates the reliability of our eQTL analyses using only the transcriptome. The Manhattan map showed a significant association between eVariants and the gene expression levels of *DYNLT1* (Figure 3A), *NMNAT1* (Figure 3B), and *MRLC2* (Figure 3C), for which 19, 20, and 7 SNPs exceeded the significant threshold, respectively. Subsequently, these results were further checked by QQ plots (Supplementary Figure S3). Meanwhile, we searched these results in the PigGTEx database and found that *NMNAT1* and *DYNLT1* have been reported, while *MRLC2* was found for the first time in this study.

### 3.3. Kmer-Based eQTL Analysis Using Transcriptome Data

**Figure 4:**
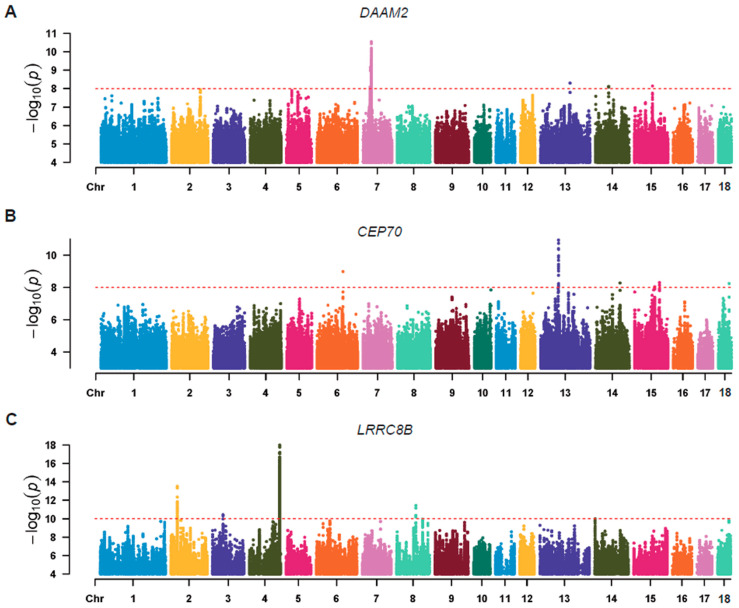
Manhattan plots of the kmer-based association results of the representative genes. Manhattan plots of (**A**) *DAAM2*, (**B**) *CEP70*, and (**C**) *LRRC8B*. The red dashed line in the Manhattan plots represents the level of significance.

Kmers with a sequence length of 31 were extracted from clean RNA sequencing data of the 102 pigs mentioned above. After a series of rigorous filtrations (see the Materials and Methods section for details), 497,926,784 unique kmers were retained for downstream analysis. Unlike previous studies that used the absence or presence of kmers as genotypes [15], we calculated the count of these unique high-quality kmers in all individuals in this study. The gene expression matrix, kinship matrix, and covariates were generated for all individuals in accordance with the process in the SNP-based eQTL analysis. In total, 35 association signals were identified at a significant threshold of 5 × 10^−8^ or a more stringent threshold of 1 × 10^−8^ via the association analysis. In order to further investigate the biological function underlying these kmers, a localization analysis of the kmers was performed using bowtie2 [32]. In total, 436,250,587 kmers were mapped in the susScr11 reference genome, accounting for 87.61%. This result again demonstrated that the kmers used in our association analysis had a high degree of credibility. By integrating the association results and localization results of the kmers, stringent filtering was performed based on the minor allele frequency, *p*-values, and mapping quality (MAPQ), and then the eQTL results of these retained kmers and the associated genes were examined. Manhattan plots showed significant associations between the expression of kmers and the gene expression levels of *DAAM2* (Figure 4A), *CEP70* (Figure 4B). where 578 and 35 kmers exceeded the set threshold (*p* value: 1 × 10^−8^), respectively. We also found kmers associated with the gene expression of *LRRC8B* (Figure 4C). By querying the PigGTEx database, it was found that all of these representative genes mined only in the kmers-based eQTLs were reported, which again demonstrates the high reliability of our study.

### 3.4. Comparison of SNP-Based and Kmer-Based eQTL Analyses

**Figure 5:**
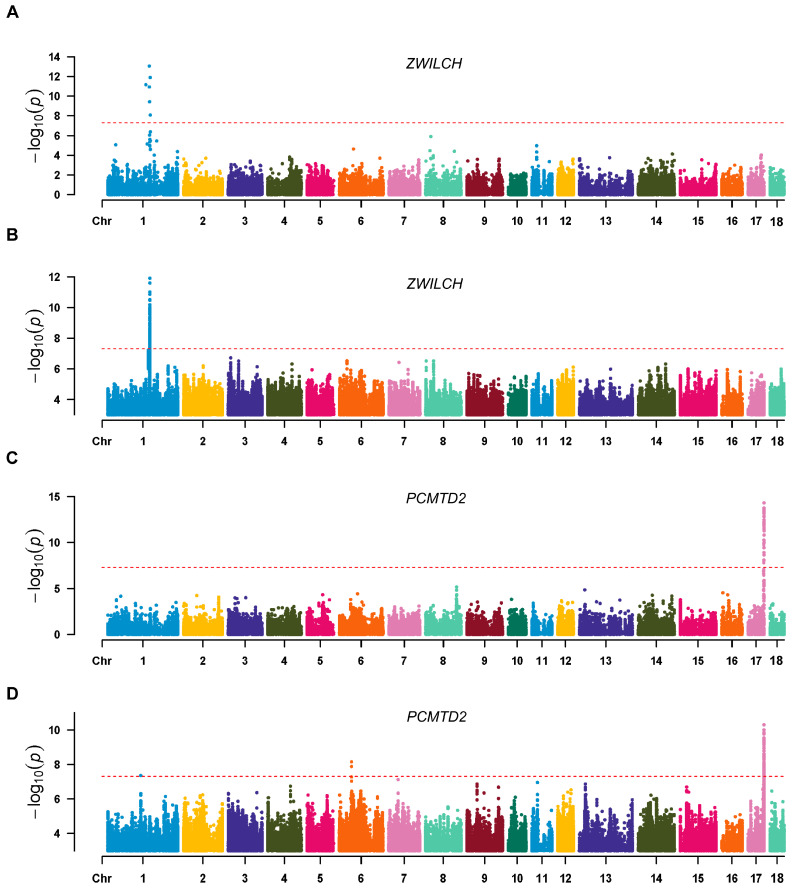
Manhattan plots of both association results of the representative genes. Manhattan plots of (**A**) *ZWILCH*, (**B**) *ZWILCH*, (**C**) *PCMTD2*, and (**D**) *PCMTD2*. The top panel shows the association results based on SNPs and the bottom panel shows the association results based on kmers. The red dashed line in the Manhattan plots represents the level of significance.

Subsequently, to determine the ability of the two approaches to identify candidate genetic variants that affect gene expression levels, the SNP-based and kmer-based association results were compared. We identified a total of 30 genes whose expression levels could be influenced by variants and kmers at the transcriptome level (Supplementary Figure S4A). This result suggests that the two approaches are complementary and also have a significant proportion of overlapped candidate signals. To further explore the biological functions of these genes associated with significant signaling loci, GO enrichment analysis was performed. We found that these genes were significantly enriched in pathways associated with actin filaments, cell cycle checkpoint signaling, and oxidoreductase activity (Supplementary Figure S4B). These pathways play indispensable roles in the development, regeneration, signaling transduction, and metabolic regulation of muscle tissue, directly or indirectly. To minimize spurious associations due to statistical power and confounding factors, we then focused on the significant association signals observed in both methods. Manhattan plots showed significant associations between variants or kmers identified from the transcriptome data and the expression levels of the genes for *ZWILCH* (Figure 5A), *PCMTD2* (Figure 5B), and *TNFRSF17* (Supplementary Figure S5). These results further indicate that our proposed methods play an important role in identifying candidate genetic variants that affect gene expression. All in all, these results show that each of the two methods has its own benefits and provide a new perspective for the mining of eQTLs.

### 3.5. Validation Analysis of Haplotype Blocks

**Figure 6:**
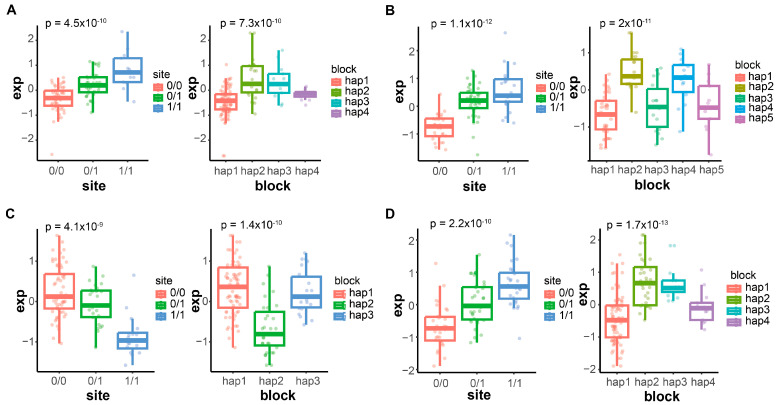
Boxplots of the validation results of the representative genes. Boxplots of (**A**) *DYNLT1*, (**B**) *NMNAT1*, (**C**) *MRLC2*, and (**D**) *PCMTD2*.

To further validate the significant association signals discovered between gene expression levels and candidate functional sites, we calculated the correlation between the top SNP sites and gene expression levels among these significant signals in all samples. In addition, in order to avoid the loss of genetic information and the weakening of statistical power captured by a single SNP locus, we extracted haplotype blocks consisting of the top SNP sites and the 10 SNP sites upstream and downstream from each, and then analyzed the correlation with the corresponding associated gene expression levels. In total, 30 hits based on SNPs alone were found, accounting for 52.63%, whereas this percentage was as high as 76.67% in the hits found by both methods (Supplementary Figure S4A). This result implies that SNPs and kmers containing multiple variant types at the transcriptome level have complementary roles in mining eQTLs. The boxplots showed the correlations between the genotype corresponding to the top SNP locus, and the genetic sequence corresponding to the haplotype block composed of its flanks and the expression levels of the associated genes (Figure 6). This result suggests that haplotype block analysis by integrating multiple SNP information may help us to better understand the genetic structure and its diversity that affects the expression levels of genes associated with economically important traits, and to improve the ability to detect true associations.

## 4. Discussion

In this study, we used only RNA-seq data to identify eQTLs by two complementary methods. Since eQTLs have broad tissue and cell type specificity [3,35], identification of eQTLs in muscle tissue is particularly important, as this is of great value for improving meat quality, increasing meat yield, and optimizing breeding strategies. Although we found more than 90% consistency between the SNPs identified by WGS and RNA-seq, the remaining fraction of variants was difficult to explain. Compared with the variants that are stably present in the genome [36], some variants in the transcriptome may occur only in specific tissues or developmental stages, which could help to discover some new eQTLs, even though these differentially variants may also have the risk of arising due to random errors. At the same time, to exclude the bias from sex chromosomes, we only retained the genetic variants on the autosomes. As a molecular phenotype, the expression level of each gene was corrected for batch effects such as the population structure, slaughter age, sex, etc., but there were inevitably other invisible confounding factors affecting the expression data from different sources.

Compared with WGS, whole-exome sequencing (WES), and SNP microarrays, RNA-seq demonstrates advantages in different aspects, such as cost savings, the availability of gene expression information, and more SNP sites, respectively. Although the cost of high-throughput sequencing is decreasing, transcriptome data for SNP calling has an indispensable value in the face of traditional GWAS studies with large samples [37]. In this study, we first performed an eQTL analysis based on SNPs identified from the transcriptome data. This method has been proposed for many years and has been widely reported and applied [38,39]. In total, we identified 87 expression variants affecting gene expression, such as *NMNAT1* (nicotinamide nucleotide adenylyl transferase 1), which has been reported to have crucial roles in the development of muscle tissues, the maintenance of physiological functions, and aging [40,41,42]. In addition, we also found genetic variants affecting the expression of *MRLC2* (myosin-regulatory light chain 2), which has been studied in the muscle tissues of hybrid offspring of different breeds of goat and pig and their parents [43,44].

As SNP calling is often highly dependent on the reference genome, we then adopted a kmer-based strategy for eQTL analysis. The generation of kmers from raw sequencing data effectively buffers the problem of genotyping that depends on the reference genome. By correlating the genotypes of kmers generated from transcriptomic data with gene expression, 35 candidate variant sites affecting gene expression level were identified, such as *DAAM2*, which has been reported to be involved in regulating the actin cytoskeleton through the wnt signaling pathway [45,46]. In addition, *LRRC8B*, found in this study, has been reported to be associated with body conformation (chest depth) in goats [47] and involved in the regulation of intracellular calcium homeostasis in humans [48]. Next, through comparing the results obtained from the two eQTL analysis methods proposed in this study, it was found that a significant proportion of genes with significantly associated signals overlapped. Meanwhile, the integrated analysis of SNP- and kmer-based eQTLs provides new perspectives for mining more genetic variant loci that are associated with complex traits or diseases by affecting gene expression. It is worth noting that only cis-eQTLs can be identified using RNA-seq data. It has to be admitted that this strategy is not suitable for mining trans-eQTLs.

Haplotype block analysis plays an essential role in improving the power of GWAS and deepening the understanding of the genetic basis of complex traits and diseases [49]. In this study, the correlation analysis of haplotype blocks and gene expression revealed that 76.67% of the association signals overlapping the results of the two eQTL analyses were validated, which was significantly higher than the validation results using only SNP-based association analysis. This further suggests that the kmer-based eQTL analysis has important application prospects in identifying candidate loci that affect gene expression. We focused on genes related to the maintenance of muscle tissue function, such as *NMNAT1* and *MRLC2*, and found that haplotype blocks consisting of the top SNP and its 10 flanking SNPs were significantly associated with gene expression. Haplotype blocks help to understand the genetic structure of populations and improve the ability to detect QTLs in GWAS by integrating information from multiple SNPs, which has been widely reported in agricultural plants and animals [1,50,51].

In the future, we will again demonstrate the robustness of our proposed new approach with larger sample sizes, in more tissues and cell types, and even in more developmental stages.

## 5. Conclusions

In this study, we adopted two different strategies for eQTL analysis. The first was to analyze the association between gene expression and SNP calling from transcriptomic data. The second was to utilize kmers generated from clean RNA-seq reads to represent variant loci and to associate these genotypes of kmers with gene expression. Subsequently, the association results obtained from these two strategies were compared and found to have a high percentage of overlap, most of which could be verified by haplotype block analysis. Additionally, this comparison also revealed that they also have the ability to mine candidate variant loci affecting gene expression. The methods of eQTL analysis utilizing only transcriptomic data can be used to mine the regulatory relationships between candidate genetic variants associated with specific traits or diseases and gene expression at a lower cost, which can help to reveal the underlying biological mechanisms to some extent.

## Data Availability

The data provided in this study can be downloaded from publicly accessible databases or requested from the corresponding author.

## Supplementary Materials

The following supporting information can be downloaded at https://www.mdpi.com/article/10.3390/ani14202941/s1, Supplementary Figures S1–S5 and Tables S1–S3 can be found in the supplementary file. Figure S1. Schematic diagram of Kmer and different genetic variants. Figure S2: Principal component analysis of variants called by the RNA sequencing data. Figure S3: Quantile–quantile plots of the associations of the representative genes. Figure S4: Comparison of the association results from the eQTL analyses based on SNPs and kmers. Figure S5: Manhattan plots of both association results for the representative genes. Table S1: Detailed information for the publicly available RNA-seq data for 102 samples. Table S2. Details of mapped reads for each sample of RNA-seq data set. Table S3. Details of mapped reads for each sample of WGS data set.
