# Supplementary material for: SNP-Based and Kmer-Based eQTL Analysis Using Transcriptome Data
Source: Animals (Basel). 2024 Oct 11;14(20):2941. doi: 10.3390/ani14202941 (PMC11503742; doi:10.3390/ani14202941)
Supplement: Supplementary file 1 [file animals-14-02941-s001.zip › animals-3239442-supplementary.pdf]

SNP based and Kmer based eQTL analysis using transcriptome data

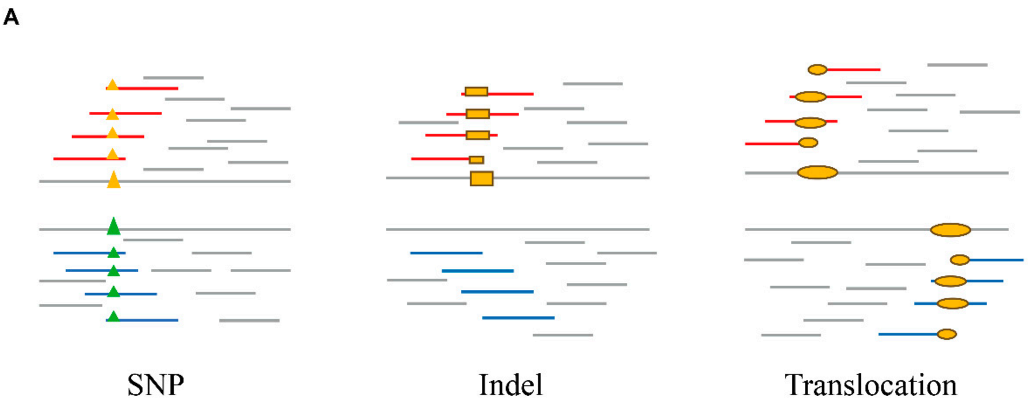

Figure S1. Schematic diagram of Kmer and different genetic variants. (A) The grey long lines indicate two individual genomes. Colored short lines refer to unique kmer for each genome, grey short lines represent shared kmer between genomes. The different shapes mark different types of genetic variation. Triangles: SNP; Boxes, Indels; Ellipses: Translocations.

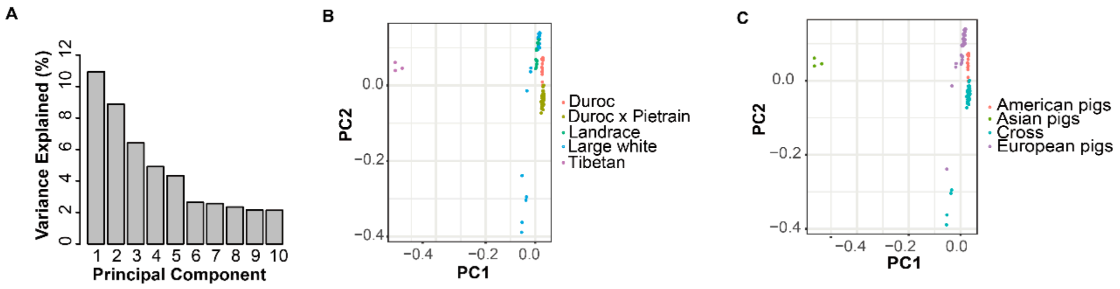

Figure S2. Principal component analysis of variants calling by RNA sequencing data. (**A**) Explanation of variance of the first ten principal components of the genotypes; (**B**) PCA plot of 102 pigs according to different breeds; (**C**) PCA plot of 102 pigs according to different breedgroups.

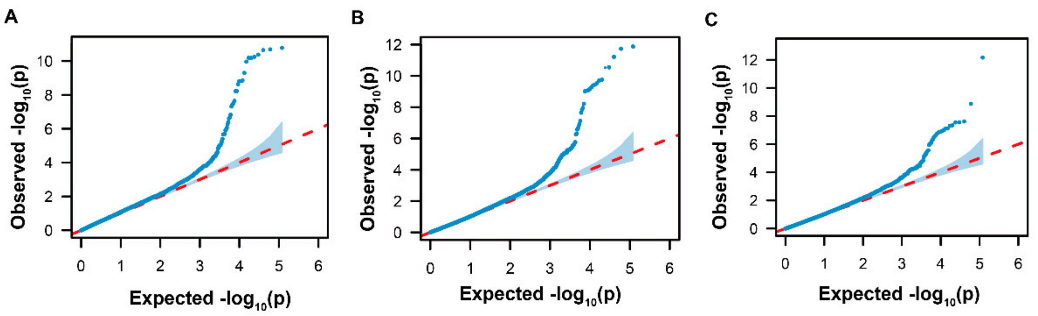

Figure S3. Quantile–quantile plots of the association results of the representative genes. Quantile–quantile plots of (**A**) *DYNLT1*; (**B**) *NMNAT1*; (**C**) *MRLC2*.

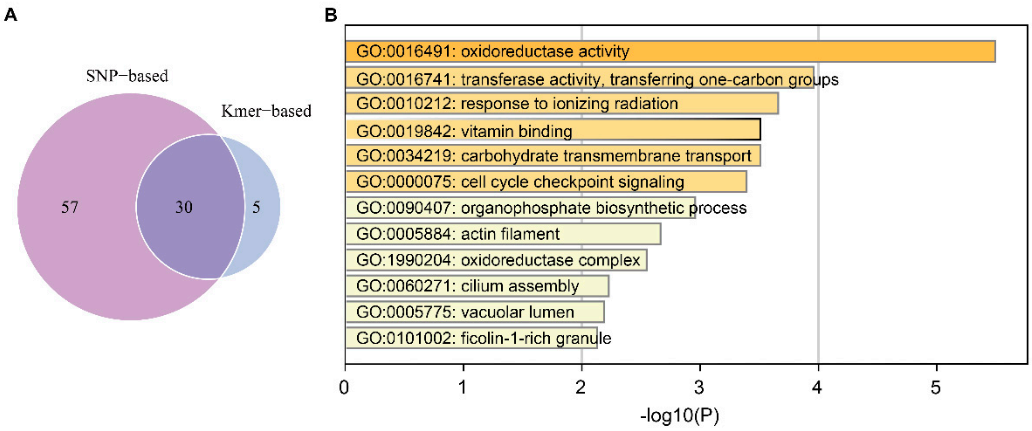

Figure S4. Comparison of association results from eQTL analysis based on SNPs and kmers. (**A**) Comparison of significant association signals found by different methods; (**B**) Go enrichment analysis of genes identified in both methods.

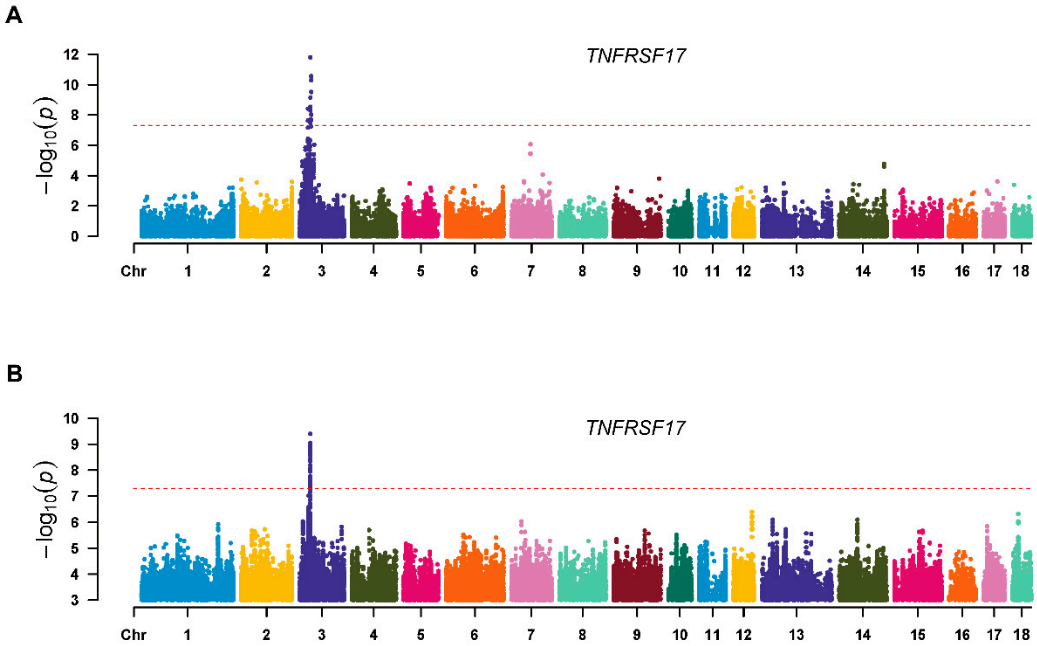

Figure S5. Manhattan plots of both association results of the representative genes. Manhattan plots of (**A**) *TNFRSF17*; (**B**) *TNFRSF17*. The top panel shows the association results based on SNPs and the bottom panel shows the association results based on Kmers. The red dashed line in the Manhattan plots represents the level of significance.

Table S1: Detailed information for the publicly available RNA-seq data for 102 samples.

BioSample	BioProject	SRR_id	Sex	Age
SAMEA4881075	PRJEB28465	ERR2775643	Male	287 days
SAMEA4881076	PRJEB28465	ERR2775644	Male	289 days
SAMEA4881077	PRJEB28465	ERR2775645	Male	276 days
SAMEA4881078	PRJEB28465	ERR2775646	Female	274 days
SAMEA4881079	PRJEB28465	ERR2775647	Female	288 days
SAMEA4881080	PRJEB28465	ERR2775648	Male	289 days
SAMEA4881081	PRJEB28465	ERR2775649	Male	287 days
SAMEA4881111	PRJEB28465	ERR2775679	Female	278 days
SAMEA4881112	PRJEB28465	ERR2775680	Male	291 days
SAMEA4881113	PRJEB28465	ERR2775681	Female	276 days
SAMEA4881114	PRJEB28465	ERR2775682	Female	275 days
SAMEA4881115	PRJEB28465	ERR2775683	Male	275 days
SAMEA4881116	PRJEB28465	ERR2775684	Female	272 days

SAMEA4881117	PRJEB28465	ERR2775685	Female	292 days
SAMEA4881118	PRJEB28465	ERR2775686	Male	276 days
SAMEA4881119	PRJEB28465	ERR2775687	Female	276 days
SAMEA4881120	PRJEB28465	ERR2775688	Male	272 days
SAMEA4881121	PRJEB28465	ERR2775689	Male	278 days
SAMEA4881122	PRJEB28465	ERR2775690	Male	0 day
SAMN05831011	PRJNA344688	SRR4299250	Male	180 days
SAMN05831013	PRJNA344688	SRR4299252	Male	180 days
SAMN05831015	PRJNA344688	SRR4299254	Male	180 days
SAMN05831034	PRJNA344688	SRR4299281	Male	180 days
SAMN05831036	PRJNA344688	SRR4299321	Male	180 days
SAMN05831038	PRJNA344688	SRR4299357	Male	180 days
SAMN07136163	PRJNA386796	SRR5617595	Female	4 weeks
SAMN07136159	PRJNA386796	SRR5617596	Female	4 weeks
SAMN07136155	PRJNA386796	SRR5617597	Female	4 weeks
SAMN07136161	PRJNA386796	SRR5617601	Female	4 weeks
SAMN07626881	PRJNA403969	SRR6023671	Female	159 days
SAMN07626884	PRJNA403969	SRR6023674	Female	158 days
SAMN07626877	PRJNA403969	SRR6023675	Female	160 days
SAMN07626963	PRJNA403969	SRR6023680	Female	163 days
SAMN07626959	PRJNA403969	SRR6023694	Female	164 days
SAMN07626956	PRJNA403969	SRR6023699	Female	164 days
SAMN07626948	PRJNA403969	SRR6023703	Female	169 days
SAMN07626952	PRJNA403969	SRR6023707	Female	165 days
SAMN07626896	PRJNA403969	SRR6023709	Female	161 days
SAMN07626888	PRJNA403969	SRR6023711	Female	158 days
SAMN07626892	PRJNA403969	SRR6023715	Female	161 days
SAMN07626893	PRJNA403969	SRR6023716	Female	161 days
SAMN07627042	PRJNA403969	SRR6023718	Female	165 days
SAMN07627041	PRJNA403969	SRR6023719	Female	165 days
SAMN07627044	PRJNA403969	SRR6023720	Male	165 days
SAMN07627043	PRJNA403969	SRR6023721	Male	165 days
SAMN07627040	PRJNA403969	SRR6023724	Male	165 days
SAMN07627039	PRJNA403969	SRR6023725	Male	165 days
SAMN07626904	PRJNA403969	SRR6023731	Female	159 days
SAMN07626900	PRJNA403969	SRR6023735	Female	159 days
SAMN07626899	PRJNA403969	SRR6023736	Male	161 days
SAMN07626906	PRJNA403969	SRR6023737	Male	159 days
SAMN07626914	PRJNA403969	SRR6023750	Female	156 days
SAMN07626911	PRJNA403969	SRR6023751	Female	157 days
SAMN07626912	PRJNA403969	SRR6023752	Male	157 days
SAMN07626910	PRJNA403969	SRR6023754	Female	157 days
SAMN07626908	PRJNA403969	SRR6023756	Female	162 days
SAMN07626916	PRJNA403969	SRR6023758	Male	156 days

SAMN07626983	PRJNA403969	SRR6023763	Male	162 days
SAMN07626981	PRJNA403969	SRR6023765	Female	162 days
SAMN07626980	PRJNA403969	SRR6023766	Male	155 days
SAMN07626920	PRJNA403969	SRR6023771	Male	156 days
SAMN07626919	PRJNA403969	SRR6023772	Female	156 days
SAMN07626917	PRJNA403969	SRR6023774	Male	156 days
SAMN07627009	PRJNA403969	SRR6023789	Female	165 days
SAMN07627007	PRJNA403969	SRR6023791	Male	165 days
SAMN07627008	PRJNA403969	SRR6023792	Male	165 days
SAMN07627013	PRJNA403969	SRR6023793	Female	165 days
SAMN07627011	PRJNA403969	SRR6023795	Male	165 days
SAMN07627012	PRJNA403969	SRR6023796	Male	165 days
SAMN07626944	PRJNA403969	SRR6023798	Female	171 days
SAMN07626975	PRJNA403969	SRR6023801	Male	156 days
SAMN07627005	PRJNA403969	SRR6023804	Female	165 days
SAMN07627004	PRJNA403969	SRR6023812	Male	165 days
SAMN07627036	PRJNA403969	SRR6023828	Male	167 days
SAMN08580364	PRJNA386796	SRR6761649	Female	4 weeks
SAMN08580363	PRJNA386796	SRR6761650	Female	4 weeks
SAMN08580371	PRJNA386796	SRR6761651	Female	4 weeks
SAMN08580370	PRJNA386796	SRR6761655	Female	4 weeks
SAMN08580369	PRJNA386796	SRR6761656	Female	4 weeks
SAMN08580362	PRJNA386796	SRR6761657	Female	4 weeks
SAMN08580368	PRJNA386796	SRR6761658	Female	4 weeks
SAMN08580367	PRJNA386796	SRR6761659	Female	4 weeks
SAMN08580361	PRJNA386796	SRR6761660	Female	4 weeks
SAMN08580366	PRJNA386796	SRR6761661	Female	4 weeks
SAMN08580372	PRJNA386796	SRR6761662	Female	4 weeks
SAMN10458223	PRJNA506343	SRR8224428	Male	1 month
SAMN10458222	PRJNA506343	SRR8224429	Female	1 month
SAMN10458221	PRJNA506343	SRR8224430	Male	1 month
SAMN10458220	PRJNA506343	SRR8224431	Female	1 month
SAMN10458217	PRJNA506343	SRR8224432	Male	1 month
SAMN10458216	PRJNA506343	SRR8224433	Female	1 month
SAMN10458219	PRJNA506343	SRR8224434	Female	1 month
SAMN10458218	PRJNA506343	SRR8224435	Male	1 month
SAMN10458213	PRJNA506343	SRR8224436	Male	1 month
SAMN10458212	PRJNA506343	SRR8224437	Male	1 month
SAMN10458215	PRJNA506343	SRR8224438	Female	1 month
SAMN10458214	PRJNA506343	SRR8224439	Male	1 month
SAMN10768518	PRJNA515765	SRR8453475	Male	2 days
SAMN10768517	PRJNA515765	SRR8453476	Male	2 days
SAMN10768516	PRJNA515765	SRR8453477	Male	2 days
SAMN10768520	PRJNA515765	SRR8453479	Male	2 days

SAMN10768519	PRJNA515765	SRR8453480	Male	2 days

Supplementary Table S2. Details of mapped reads for each sample of RNA-seq data set.

Sample	Total_reads	Mapped_reads	paired_reads	Properly_paired reads	Average depth	Coverage
20101	241273144	241273144	222699362	222699362	39.25	58.87%
20203	232557648	232557648	201598420	201598420	34.03	55.32%
5572	285672812	285672812	259710276	259710276	26.14	54.13%
5814	280820526	280820526	251535678	251535678	33.72	54.56%
M15S1	283377046	283377046	257128096	257128096	32.36	54.03%
M15S2	204415812	204415812	194577000	194577000	27.2	42.39%

Supplementary Table S3. Details of mapped reads for each sample of WGS data set.

Sample	Total_reads	Mapped_reads	paired_reads	Mapping rate	Properly_paired reads	Properly_paired rate
20101	1135765878	1131498958	1129826564	99.62%	1100019916	97.36%
20203	1017545006	1013747986	1011994016	99.63%	984133050	97.25%
5572	902419743	898542715	896099466	99.57%	867759676	96.84%
5814	940872887	937109031	935174564	99.60%	908554922	97.15%
M15S1	1073465408	1069660650	1067262750	99.65%	1037191148	97.18%
M15S2	832588720	829174251	825677446	99.59%	794807148	96.26%
